# Effect of Pr in CO_2_ Methanation Ru/CeO_2_ Catalysts

**DOI:** 10.1021/acs.jpcc.1c03539

**Published:** 2021-05-27

**Authors:** Sergio López Rodríguez, Arantxa Davó-Quiñonero, Jerónimo Juan-Juan, Esther Bailón-García, Dolores Lozano-Castelló, Agustín Bueno-López

**Affiliations:** † Inorganic Chemistry Department, University of Alicante, Carrertera de San Vicente del Raspeig s/n, E-03080 Alicante, Spain; ‡ School of Chemistry, CRANN and AMBER Research Centres, Trinity College Dublin, College Green, Dublin 2, Dublin, Ireland; § Technical Research Services (SSTTI), University of Alicante, Carretera de San Vicente del Raspeig s/n, E-03080 Alicante, Spain

## Abstract

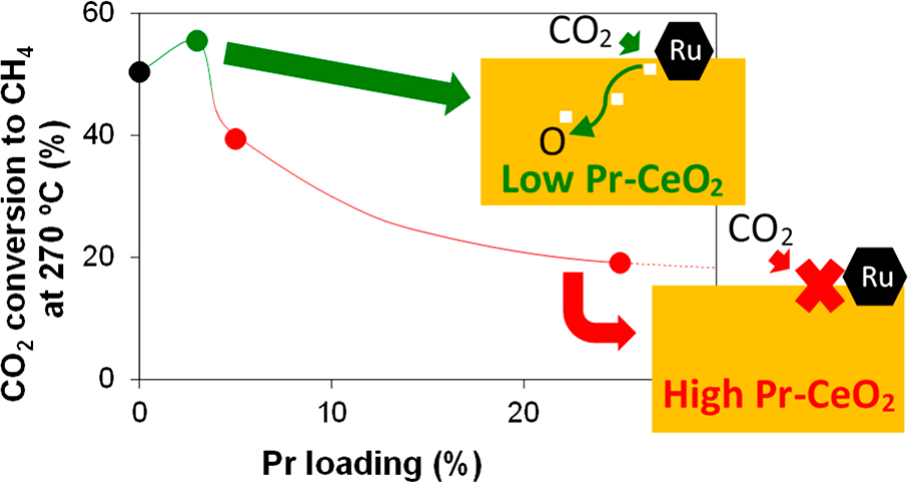

CO_2_ methanation has been studied with Pr-doped Ru/CeO_2_ catalysts, and a dual effect of Pr has been observed. For low Pr content (i.e., 3 wt %) a positive effect in oxygen mobility prevails, while for high Pr doping (i.e., 25 wt %) a negative effect in the Ru–CeO_2_ interaction is more relevant. Isotopic experiments evidenced that Pr hinders the dissociation of CO_2_, which takes place at the Ru–CeO_2_ interface. However, once the temperature is high enough (200 °C), Pr improves the oxygen mobility in the CeO_2_ support, and this enhances CO_2_ dissociation because the oxygen atoms left are delivered faster to the support sink and the dissociation sites at the interface are cleaned up faster. In situ Raman spectroscopy experiments confirmed that Pr improves the creation of oxygen vacancies on the ceria lattice but hinders their reoxidation by CO_2_, and both opposite effects reach an optimum balance for 3 wt % Pr doping. In addition, in situ diffuse reflectance infrared Fourier transform spectroscopy (DRIFTS) experiments showed that Pr doping, regardless of the amount, decreases the population of surface carbon species created on the catalysts surface upon CO_2_ chemisorption under methanation reaction conditions, affecting both productive reaction intermediates (formates and carbonyls) and unproductive carbonates.

## 1 Introduction

Cerium oxide materials have relevant utility in heterogeneous catalysts and have been the center of a significant battery of studies devoted to gaining insight into their high performance. The unique properties of ceria rely on its oxygen storage/release capacity (OSC) and strong synergistic metal–support interactions when a metal phase is dispersed on a ceria matrix.^1−3^ Not only promoting their catalytic activity but also understanding the effect on these active sites will lay the foundations of a rational design of heterogeneous catalysts.

CeO_2_ doping with other cations usually improves the catalytic features due to the creation of oxygen vacancies,^4−14^ but the beneficial effect is often obtained only when the content is optimized. It is well-known that doping ceria with Zr^4+^, Ti^4+^, Eu^3+^, La^3+^, or Tb^3+^ cations, among others, improves the catalytic activity in different reactions, such as volatile organic compound (VOC) oxidation, CO oxidation, soot combustion, and NOx reduction.^5−7,14,15^ Ceria–praseodymia catalysts have demonstrated superior redox properties and improved catalytic performance compared to other ceria-based mixed oxides in soot combustion and CO oxidation reactions, and these Ce–Pr formulations are currently under investigation in other catalytic applications.^16−20^

CO_2_ conversion to methane by H_2_ is a practical strategy for clean energy utilization with the dual benefit of reducing CO_2_ emissions while meeting the increasing energy demand forecasted for the next few decades.^21,22^ Metals supported on ceria can activate CO_2_ molecules at lower temperatures compared to analogue catalysts with other supports. Ceria improves the efficiency of the reaction because oxygen vacancies serve as active sites for CO_2_ dissociation.^23−27^ Up to now, the research on this reaction has shed light on the catalytic role of each active center by means of in situ spectroscopies and other advanced techniques.^28−34^ It is known that CO_2_ methanation requires a bifunctional catalyst able to undertake both H_2_ and CO_2_ activation processes on one or two different type of sites. In heterogeneous catalysts based on inert supports (such as Al_2_O_3_ or SiO_2_), both events take place on reduced metal sites.^35−40^ However, ceria-based CO_2_ methanation catalysts are proven to be more efficient because ceria contributes to CO_2_ chemisorption and dissociation in cooperation with the metal. That is, H_2_ dissociation takes place preferentially on reduced metal sites while the most efficient sites for CO_2_ dissociation are located at the metal–ceria interface.^28,41^

This study focuses on the investigation of the role of Pr in Ru/CeO_2_ catalyst for CO_2_ methanation by analyzing the effect of Pr loading. Pr is able to adopt +3 and +4 oxidation states, like Ce cations, and Pr cations can be introduced into the fluorite CeO_2_ lattice, forming a solid solution until ∼30% Pr (with regard to Ce + Pr), with segregated Pr_6_O_11_ being expected above this threshold.^42^

In this study, in situ Raman and in situ diffuse reflectance infrared Fourier transform spectroscopy (DRIFTS) experiments have been performed for the assessment of the role of oxygen vacancies and the identification of reaction intermediates under reaction conditions. Steady-state isotopic exchange (SSIE) experiments have been performed to study the oxygen exchange between CO_2_ and the catalysts by evaluating the activation of the CO_2_ molecules on the Pr-doped Ru/CeO_2_ catalysts.

## 2 Experimental Details

### 2.1 Catalyst Preparation

Ruthenium catalysts with Pr-doped CeO_2_ supports, referred to as Ru/Ce%PrO_*x*_ onward, were prepared and used in this study. The “%” corresponds to the weight percent of Pr with regard to Ce + Pr, which ranges from 0 to 25%. A Ru/PrO_*x*_ catalyst was also prepared.

The necessary amounts of Ce(NO_3_)_3_·6H_2_O (99%, Sigma-Aldrich) and Pr(NO_3_)_3_·6H_2_O (99.9%, Sigma-Aldrich) were physically mixed in a mortar and calcined at 600 °C during 6 h. Nitrates decompose to oxides during calcination and Ce^3+^ and Pr^3+^ cations were oxidized to a 4+ oxidation state, forming a Ce_*x*_Pr_1–*x*_O_2_ solid solution. Ruthenium was loaded afterward by incipient wetness impregnation of the corresponding amount of ruthenium(III) acetylacetonate (97%, Sigma-Aldrich) to achieve 3 wt % Ru on the final catalysts. Finally, the impregnated samples were thermally treated at 350 °C during 3 h in N_2_ atmosphere, using a heating ramp of 5 °C/min.

### 2.2 Catalysts Characterization

The ruthenium content was determined by inductively coupled plasma–optical emission spectrometry (ICP-OES) in a PerkinElmer device (Optima model 4300 DV) after digestion of the catalysts in a HCl/HNO_3_ (3:1 volume) mixture assisted by microwaves.

The crystalline structure of each catalyst was analyzed by X-ray diffraction in a Rigaku Miniflex II diffractometer. The diffractograms were recorded in a range of 2θ from 10° to 90°, with a step of 0.025°. The wavelength used was λ = 0.155418 nm, corresponding to the Cu Kα radiation. The average crystal size of ceria was determined using the Scherrer equation. N_2_ physisorption isotherms were measured at −196 °C in an Autosorb-6 device (Quantachrome) after outgassing the catalysts during 4 h at 250 °C under vacuum conditions.

The reducibility of the catalysts was examined by H_2_ temperature-programmed reduction (H_2_-TPR) in a Micromeritics Pulse Chemisorb 2705 device. For such measurements, 25 mg of catalyst was loaded in a tubular quartz reactor coupled with a thermal conductivity detector (TCD) while flowing a mixture consisting of 40 mL/min of 5% H_2_/Ar. The temperature was increased at a 10 °C/min pace from room temperature up to 950 °C.

### 2.3 Catalytic Tests

Catalytic tests were performed in a fixed-bed tubular reactor (10 mm inner diameter) containing 100 mg of catalyst mixed with SiC particles (1.00–1.25 mm) to reach a bed volume of 1 cm^3^. The catalyst was pretreated in situ at 500 °C for 1 h under 100 mL/min of a 50% vol H_2_/He mixture. After cooling down to room temperature, the reaction mixture was fed to the reactor. The feed consisted of 100 mL/min of 10% CO_2_, 40% H_2_, and He balance at 1 atm. The GHSV was 9 000 h^–1^. The outlet gases were monitored under steady-state conditions at each temperature with a gas chromatograph (Agilent 8860 GC System) equipped with two packed columns (Porapak Q 80/100 for CO_2_ and Molecular Sieve 13× for O_2_ and CO separation) coupled to a TCD.

### 2.4 Isotopic Experiments

Steady-state isotopic experiments were performed with ^13^C^18^O_2_ (Aldrich; 99% ^13^C, 95% ^18^O) pulses in a fixed-bed cylindrical reactor with 4 mm inner diameter coupled to a Pfeiffer Vacuum mass spectrometer (model OmniStar) operating at 1 ms frequency. The catalytic bed (50 mg) was pretreated under 20 mL/min of 50% H_2_/He mixture at 500 °C for 1 h and then cooled down to room temperature under He atmosphere. The reaction mixture (10% CO_2_, 40% H_2_, and He balance) was then continuously fed to the reactor (20 mL/min), and different gas pulses were fed to this main stream. A six-way valve with a loop of 100 μL was used and was filled with 9 psi of the gas to be pulsed. This volume of gas was dragged by the main gas stream once the position of the valve was changed. One Ar pulse followed by a pulse of isotopic ^13^C^18^O_2_ was fed at 25, 100, 200, 250, and 300 °C.

### 2.5 In Situ Raman Spectroscopy Experiments

In situ Raman spectra were recorded in a LabRam Jobin Ivon Horiba instrument with a laser excitation source of He/Ne (632.8 nm). Experiments were performed in a high-temperature chamber fed with a gas flow of 60 mL/min. The catalysts were reduced with 50% H_2_/N_2_ for 1 h at 450 °C, and then the catalysts were cooled down to room temperature under N_2_ flow. The methanation gas mixture (10% CO_2_, 40% H_2_, and N_2_ balance) was fed to the cell, and spectra were recorded in steady state at 25, 100, 200, 300, and 400 °C. A monocrystalline Si reference (521 cm^–1^) was used to calibrate the position of the bands.

### 2.6 In Situ DRIFTS Experiments

In situ DRIFTS experiments were performed in a Jasco infrared spectrometer, model FT/IR-4000, using a reaction cell with temperature and gas flow control. The gas composition was monitored during the experiments with a Pfeiffer Vacuum mass spectrometer (model OmniStar). The catalytic bed consisted of 90 mg of catalyst, which was pretreated in 50% H_2_/He at 450 °C for 1 h and then cooled down to room temperature under He atmosphere. A background spectrum was recorded in these conditions, and then 100 mL/min of the methanation mixture (10% CO_2_, 40% H_2_, and He balance) was fed. Spectra were recorded from 4000 to 1000 cm^–1^ in steps of 1 cm^–1^ at 25, 100, 200, 300, and 350 °C once steady-state conditions were achieved at each temperature.

## 3 Results and Discussion

### 3.1 Catalytic Tests

**Figure 1:**
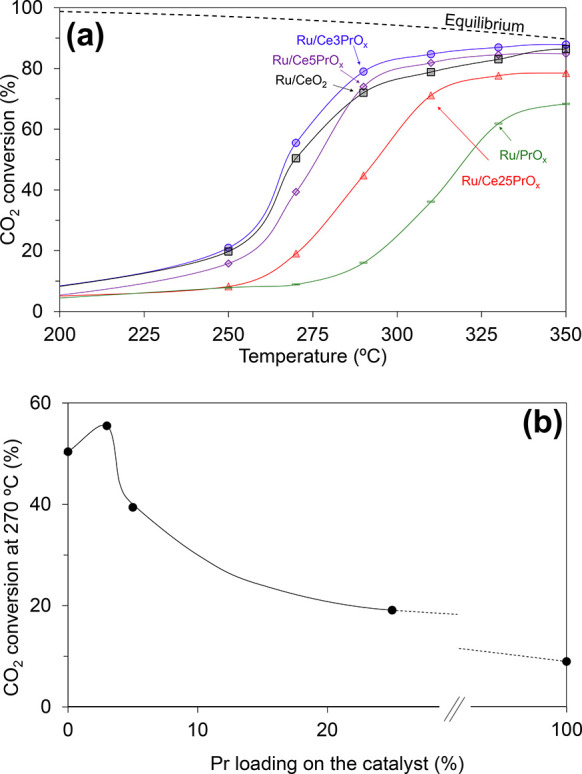
CO_2_ methanation experiments performed in a fixed-bed reactor under steady-state conditions using Ru catalysts with different LnO_*x*_ supports (Ln = Ce and/or Pr). Reduction pretreatment at 500 °C for 1 h in 50% H_2_/He. Reaction mixture: 10% CO_2_, 40% H_2_, and He balance. (a) CO_2_ conversion for different temperatures and (b) CO_2_ conversion at 270 °C for different Pr loadings.

Figure 1a shows the CO_2_ conversion to methane as a function of the temperature obtained in the catalytic experiments, and Figure 1b presents the conversion CO_2_ at a selected temperature (270 °C) as a function of the Pr loading of the catalysts. All catalysts displayed 100% selectivity to methane, which is a feature that can be attributed to ruthenium catalysts,^43−47^ and the catalytic activity observed follows this trend:
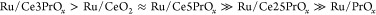
This trend indicates that Pr doping in low concentration is beneficial, with Ru/Ce3PrO_*x*_ being moderately more active than Ru/CeO_2_ from 250 °C onward, while high Pr concentration has a negative effect. Taking these results into account, three catalysts (Ru/Ce3PrO_*x*_, Ru/Ce25PrO_*x*_, and Ru/CeO_2_) were selected to conduct an extended investigation of the effect of Pr in the catalytic behavior of Ru/CeO_2_ catalysts.

### 3.2 ICP, X-ray Diffraction, and N_2_ Adsorption Characterization

The Ru loading was determined by ICP, and the values obtained are included in Table 1. The Ru content was below the nominal target value (3%) for all catalysts, probably because part of the metal was released during the thermal treatment due to the high volatility of some RuO_*x*_ species.^48^ The thermal treatment performed to decompose the Ru precursor salt was carried out under inert atmosphere in order to minimize this effect, but the formation of RuO_*x*_ volatile species with the oxygen available in the metal precursors cannot be ruled out. It is known that ceria interacts strongly with Ru species and diminishes the release of volatile RuO_*x*_ oxides with regard to other supports, such as SiO_2_ or Al_2_O_3_.^40−49^ This effect would explain the lower Ru content determined for the Ru/Ce25PrO_*x*_ catalyst (2.1% of Ru) with regard to that measured for the catalyst without Pr (2.5% for Ru/CeO_2_) and with little Pr (2.6% for Ru/Ce3PrO_*x*_) because Pr doping partially hinders the RuO_*x*_–CeO_2_ interaction, as will be demonstrated later. The effect of Pr doping in the Ru content is relevant for 25% Pr doping but not for 3% Pr doping.

**Table 1:** Results of Catalysts Characterization by ICP-OES, X-ray Diffraction (XRD), and N_2_ Adsorption–Desorption

	Ru content (wt %)	lattice parameter of ceria (nm)	crystallite size of ceria (nm)	BET specific surface area (m^2^/g)
Ru/CeO_2_	2.5	0.540	13	90
Ru/Ce3PrO_*x*_	2.6	0.542	13	40
Ru/Ce25PrO_*x*_	2.1	0.543	16	70

**Figure 2:**
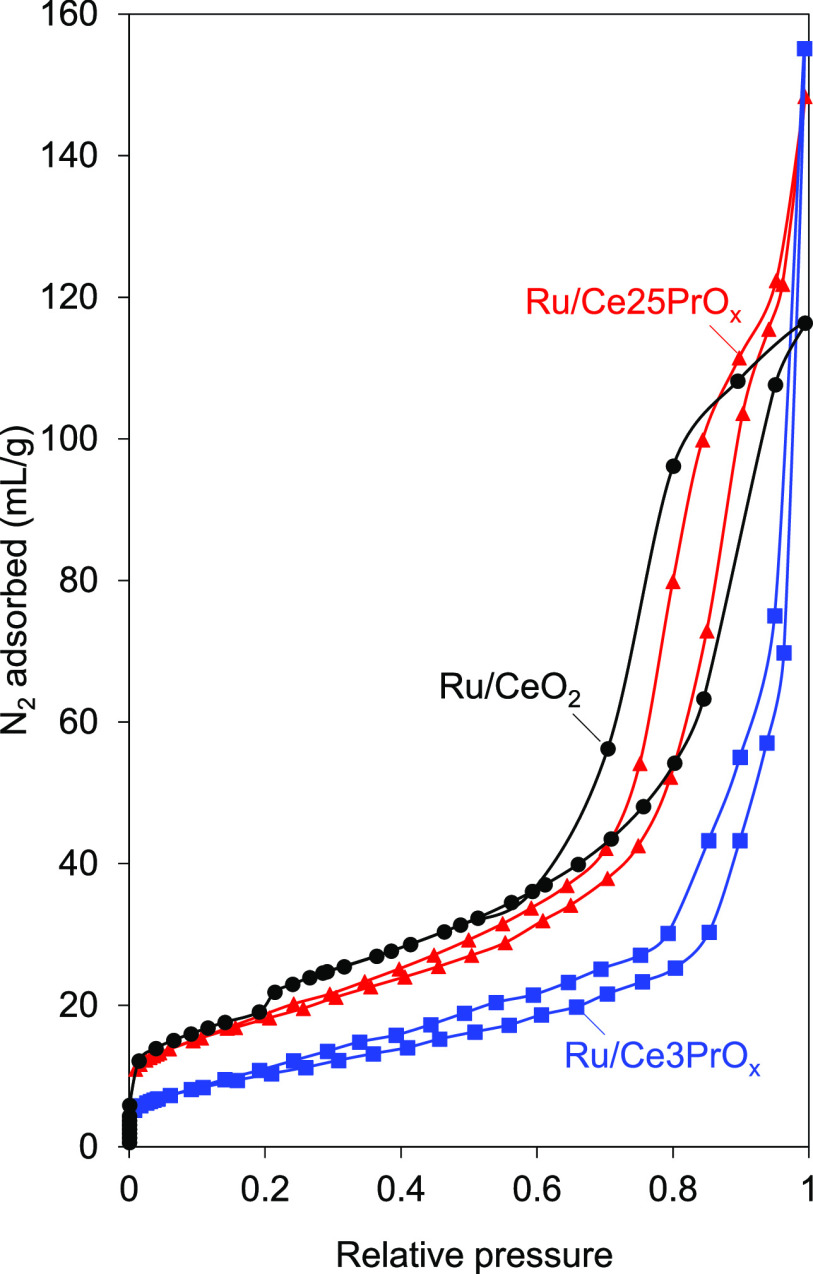
N_2_ adsorption–desorption isotherms of the catalyst.

The catalyst porosity was studied by N_2_ adsorption–desorption, and the isotherms at −196 °C are shown in Figure 2.

All isotherms show certain adsorption at low partial pressure due to the presence of narrow porosity followed by a hysteresis loop at higher partial pressures attributed to meso- and/or macropores. Pr doping changes the shape of the hysteresis loop, whose type indicates, for the Ru/CeO_2_ catalyst, the presence of mesopores, while the absence of a plateau in the isotherms of Ru/Ce3PrO_*x*_ and Ru/Ce25PrO_*x*_ evidences the formation of macropores. The Brunauer–Emmett–Teller (BET) specific surface areas, which are included in Table 1, range between 90 and 40 m^2^/g, and these values are consistent with those reported in the literature for similar materials. As shown in Table 1, the Pr-containing catalysts present a lower surface area compared to Ru/CeO_2_ due to the promoting effect of the Pr cations in CeO_2_ sintering. Usually, CeO_2_ doping with foreign cations favors sintering during calcination for moderate temperatures (∼500–700 °C) because dopants create defects on the ceria lattice that decrease the energy barrier that must be overcome for crystals to grow. On the contrary, dopants partially avoid sintering for high calcination temperatures where the energy barrier for sintering is already overcome.

**Figure 3:**
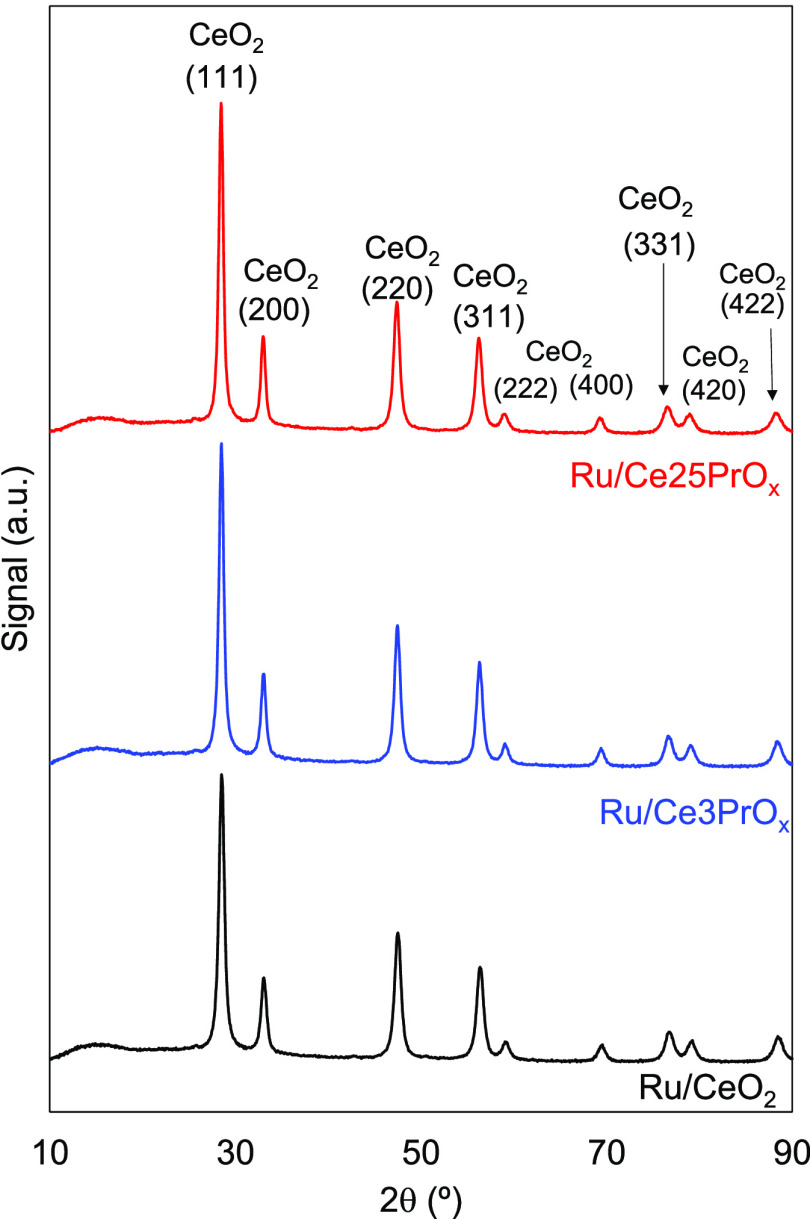
XRD patterns of the catalysts.

The crystalline structure of the catalysts was characterized by XRD, and the diffractograms are compiled in Figure 3. As expected, all catalysts show diffractograms characteristic of the fluorite structure of ceria (JCPDS file 34-0394). Evidence of ruthenium species was not found in the diffractograms, which indicates that ruthenium species are highly dispersed on the ceria support.

Identification of PrO_*x*_ phases in CeO_2_-rich oxides is difficult by XRD because their diffraction patterns are analogous. Pr can adopt several PrO_*x*_ stoichiometries with *x* ≤ 2, and their diffraction patterns are only slightly different from that of ceria (JCPDS file 24-1006 for PrO_2_ and 42-1121 for Pr_6_O_11_, for instance). In addition, Pr cations can be easily inserted into the CeO_2_ matrix. Evidence of segregated PrO_*x*_ phases can be observed in some cases as shoulders in the CeO_2_ peaks, but these are not obvious in Figure 3, suggesting the formation of solid solutions with Pr cations inserted in the CeO_2_ framework and/or the presence of highly dispersed PrO_*x*_ phases spread on the ceria surface.

The lattice parameter and crystallite size of the CeO_2_ phase were determined, and the calculated values are compiled in Table 1. The lattice parameter is consistent with that reported for pure ceria (ca. 0.540 nm) for all catalysts, and Pr doping does not significantly affect this value.^18,37^ This does not rule out the insertion of Pr cations into the ceria lattice because the sizes of Ce and Pr cations are similar and neither expansion nor contraction of the ceria lattice is expected upon Pr doping. The calculated crystallite sizes are also similar for the three catalysts (13–16 nm), evidencing that Pr doping has only a moderate effect on the size of the primary crystals.

### 3.3 H_2_ Temperature-Programmed Reduction

**Figure 4:**
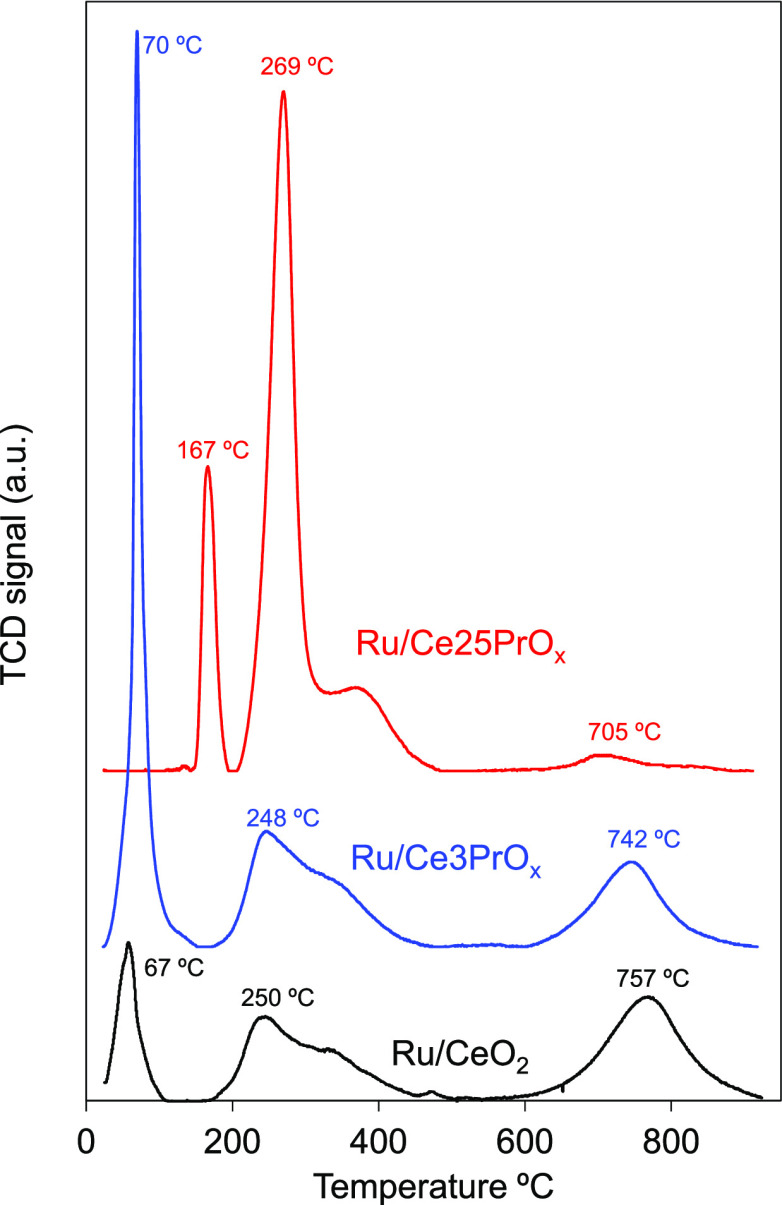
H_2_-TPR profiles of the catalysts.

The reducibility of the catalysts was studied by H_2_-TPR, and the reduction profiles are shown in Figure 4. Three reduction peaks can be distinguished in all reduction curves, but the temperature of each event depends on the nature of the catalyst. The consumption of H_2_ on these peaks has been quantified, and the values are compiled in Table 2.

**Table 2:** H_2_ Consumed in H_2_-TPR Experiments^a^

catalyst	lowest-temperature peak, mmol H_2_/mmol Ru	intermediate-temperature peak, mmol H_2_/mmol Ln	highest-temperature peak, mmol H_2_/mmol Ln
Ru/CeO_2_	1.04	0.07	0.08
Ru/Ce3PrO_*x*_	2.74	0.11	0.07
Ru/Ce25PrO_*x*_	1.46	0.30	0.02

a Ln = Ce + Pr.

The lowest-temperature peaks at 67, 70, and 167 °C for Ru/CeO_2_, Ru/Ce3PrO_*x*_, and Ru/Ce25PrO_*x*_, respectively, are mainly attributed to RuO_*x*_ reduction to Ru. According to the literature,^43,45^ ruthenium is expected to mainly form RuO_2_ in these types of materials, and the mol of H_2_ consumed in the lowest-temperature peak for the Ru/CeO_2_ catalyst is half of that required for the total reduction of RuO_2_ to Ru. On the contrary, the amount of H_2_ consumed in the lowest-temperature peak by the Ru/Ce3PrO_*x*_ catalyst exceeds the amount required for 100% RuO_2_ reduction, suggesting that Ce^4+^ and/or Pr^4+^ cations are being reduced together with those of ruthenium at this low temperature. This result evidences the enhanced reducibility of the Ru/CeO_2_ catalyst upon 3% Pr doping. However, the opposite effect of Pr is observed for 25% doping because the lowest-temperature reduction peak of Ru/Ce25PrO_*x*_ is delayed to 167 °C, and the consumption of H_2_ decreases significantly with regard to Ru/Ce3PrO_*x*_.

The intermediate- and highest-temperature peaks must be mainly assigned to the reduction of Ce^4+^ and Pr^4+^ cations together with the reduction of the remaining RuO_*x*_ species, the former to surface reduction and the latter to bulk reduction. The presence of shoulders in these peaks evidences the overlapping of several events, which can be attributed to the slightly different reducibilities of the Ce^4+^ and Pr^4+^ cations and to the presence of different surface species on the catalysts, including hydroxyl groups, carbonates/bicarbonates, etc., that can be reduced or desorbed and methanated during the heating reduction.

Surface reduction of bare ceria usually starts around 400 °C, but the surface reduction peak is shifted to 250 °C in the Ru/CeO_2_ catalyst due to the catalytic effect of Ru in the reduction of ceria. Ruthenium species, once reduced to a metal state, are very efficient for H_2_ dissociation, accelerating the reduction of the support. Quantification of H_2_ consumed in this intermediate peak indicates that Pr doping enhances the reduction of the support; the higher the Pr loading, the higher is the achieved support surface reduction degree.

The improved reducibility of the support upon Pr doping is also observed in the highest-temperature peak. The temperature of this bulk reduction peak is shifted from 757 °C for Ru/CeO_2_ to 742 °C for Ru/Ce3PrO_*x*_ as a consequence of the improved reducibility of the support in the latter catalyst, but it roughly keeps the same reduction degree (0.08–0.07 mmol H_2_/mmol Ln). On the contrary, the bulk reduction peak almost disappears for Ru/Ce25PrO_*x*_, while the area of the intermediate-temperature peak increases drastically. This behavior occurs because of the high oxygen mobility into the 25% Pr-doped CeO_2_ lattice because bulk oxygen is pumped out to the surface with a low energy barrier once the surface oxygen is depleted.

As a summary, these H_2_-TPR experiments allow the conclusion that Pr doping significantly improves the reducibility and oxygen mobility of the ceria support. In addition, RuO_*x*_ reduction is improved for low Pr loading (3%), while it is impeded for high Pr loading (25%).

### 3.4 Steady-State Isotopic Exchange Experiments

To analyze the effect of Pr in the interaction of CO_2_ with the Ru/CeO_2_ catalysts, steady-state isotopic exchange (SSIE) experiments were carried out using isotopic CO_2_. Table 3 compiles the carbon species and mass spectroscopy signals involved in these changes to facilitate the interpretation of the experiments.

**Table 3:** Carbon Species and *m*/*z* Signals Involved in the Isotopic Experiments

*m*/*z*	species	isotopic composition
15	CH_4_	^12^CH_4_
16	CH_4_	^13^CH_4_
44	CO_2_	^12^C^16^O^16^O
45	CO_2_	^13^C^16^O^16^O
46	CO_2_	^12^C^16^O^18^O
47	CO_2_	^13^C^16^O^18^O
48	CO_2_	^12^C^18^O^18^O
49	CO_2_	^13^C^18^O^18^O

Isotopic CO_2_ (^13^C^18^O^18^O; *m*/*z* 49) was pulsed at different temperatures to a methanation mixture with 10% CO_2_ (^12^C^16^O^16^O; *m*/*z* 44) + 40% H_2_ in He balance, which was continuously fed to the reactor. The oxygen and carbon species present in the catalysts before the isotopic pulses only include ^16^O and ^12^C atoms, and therefore, the release of species with ^18^O and ^13^C atoms provides information about the reaction pathway followed by the CO_2_ (^13^C^18^O^18^O) molecules pulsed. Pulse experiments performed without catalyst confirmed that no gas-phase reactions take place in the experiment conditions. Note also that CO species were not detected in the isotopic experiments, in accordance with the high selectivity of these catalysts toward CH_4_. H_2_^18^O (*m*/*z* 20) was not detected in any isotopic experiments regardless of the catalyst and temperature, that is, all oxygen atoms in H_2_O emitted as a product of the methanation reaction are nonisotopic (^16^O).

**Figure 5:**
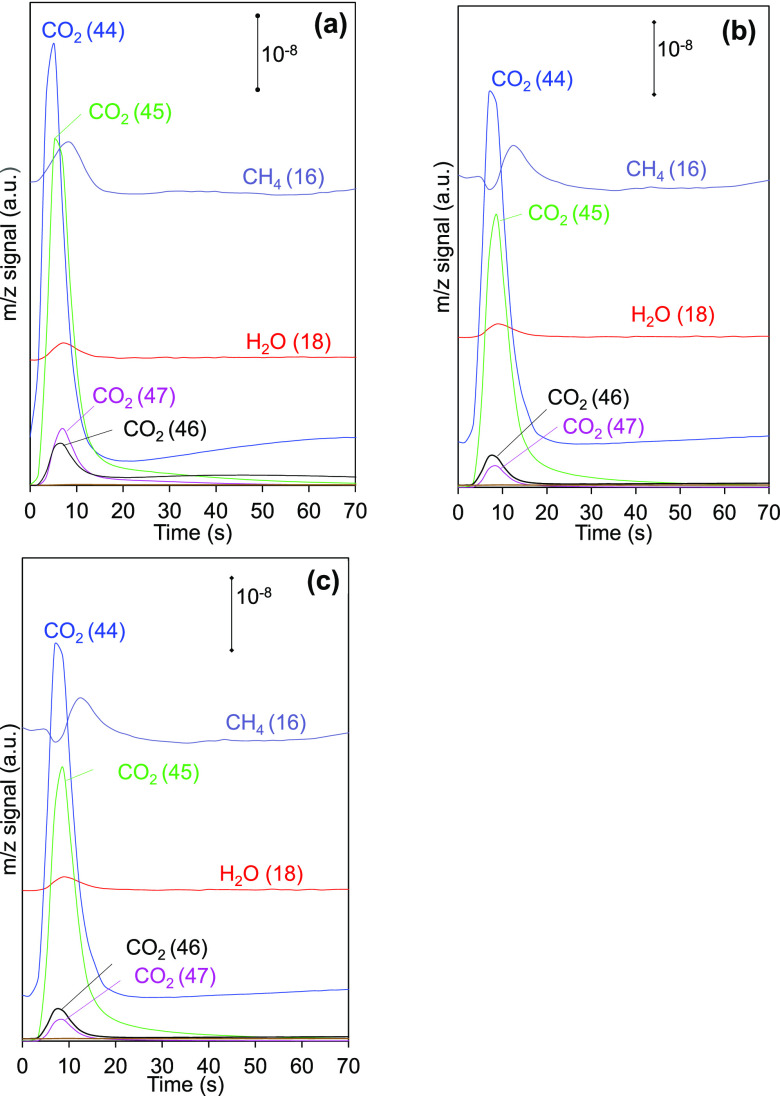
Main signals detected upon CO_2_ (^13^C^18^O^18^O; *m*/*z* 49) pulses at 250 °C to the different catalysts under CO_2_ methanation conditions (10% ^12^C^16^O^16^O (*m*/*z* 44) + 40% H_2_ in He). (a) Ru/CeO_2_, (b) Ru/Pr3CeO_*x*_, and (c) Ru/Pr25CeO_*x*_. *x*-axis origin (0 s) corresponds to the time of the pulse.

Figure 5 shows, as a representative example, the main signals recorded upon a CO_2_ (^13^C^18^O^18^O; *m*/*z* 49) pulse at 250 °C for each catalyst. The *m*/*z* 49 signal is not detected in the conditions of Figure 5, which indicates that pulsed CO_2_ is stored on the catalysts or transformed to other species. The products of the methanation reaction (CH_4_ and H_2_O) are observed, as well as several CO_2_ species including ^12^C^16^O^16^O (*m*/*z* 44), ^13^C^16^O^16^O (*m*/*z* 45), ^12^C^16^O^18^O (*m*/*z* 46), and ^13^C^16^O^18^O (*m*/*z* 47). This evidences that CO_2_ molecules are involved in a dynamic equilibrium between the gas phase and the catalyst surface, which results in the activation of the CO_2_ molecules. This activation coupled with the oxygen exchange capacity yields different carbon species with *m*/*z* signals between 45 and 48. As such, the obtained reaction products (CH_4_ and H_2_O) are released in the form of isotopic (^13^CH_4_), nonisotopic (^12^CH_4_ and H_2_^16^O), or a combination of them. Note that the isotopic methane peak is the result of the hydrogenation of carbon species coming from the CO_2_ (^13^C^18^O^18^O; *m*/*z* 49) pulse, and the release of nonisotopic methane (^12^CH_4_; *m*/*z* 15) indicates that the chemisorption of new species upon the CO_2_ (^13^C^18^O^18^O; *m*/*z* 49) pulse promotes the hydrogenation of carbon species previously present on the catalysts.

**Figure 6:**
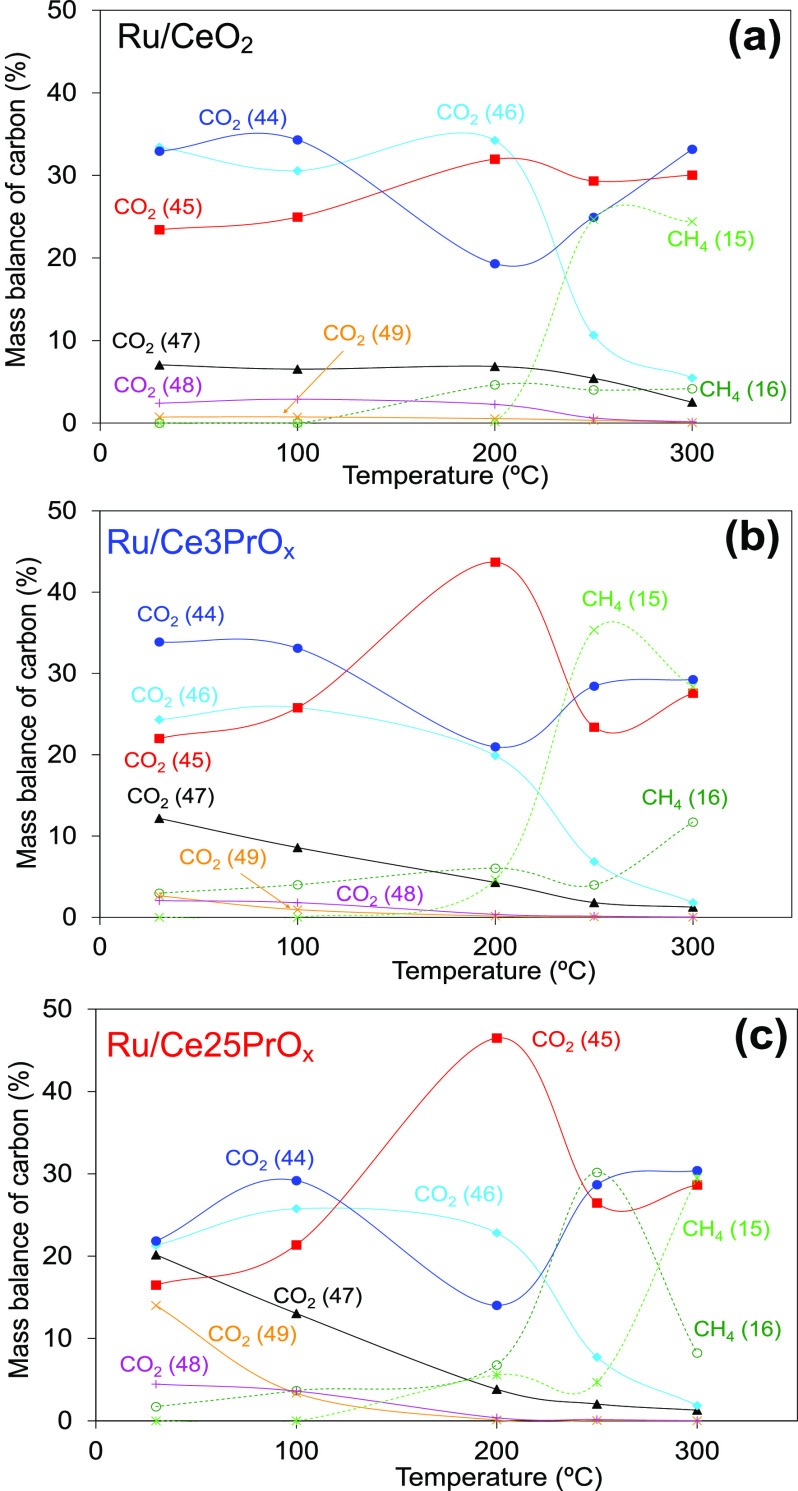
Mass balance of carbon species upon CO_2_ (^13^C^18^O^18^O; *m*/*z* 49) pulses at different temperatures under CO_2_ methanation gas flow. (a) Ru/CeO_2_, (b) Ru/Ce3PrO_*x*_, and (c) Ru/Ce25PrO_*x*_. Pretreatment of 50% H_2_/He at 450 °C, 1 h; methanation mixture 10% ^12^C^16^O_2_, 40% H_2_, and He balance.

Figure 5 allows for the conclusion that the qualitative behaviors of the three catalysts are similar, that is, the types of species evolved are the same for all catalysts. Nevertheless, quantitative differences can be distinguished that are attributed to Pr doping. The quantification of the areas under the different signals in the pulse experiments let us determine the mass balance of carbon in each pulse (Figure 6). It was considered for those calculations that the system is in steady state, and therefore, there is no net accumulation of carbon species on the catalyst surface after a single pulse.

As expected, reaction products (CH_4_ and H_2_O) were not detected at 30 °C for any catalyst, in accordance with the catalytic experiments shown in Figure 1, and the species released after the CO_2_ (^13^C^18^O^18^O; *m*/*z* 49) pulse at this temperature were CO_2_ molecules in a diverse isotopic combination. This means that the catalysts are able to chemisorb isotopic CO_2_ (^13^C^18^O^18^O; *m*/*z* 49) gas molecules, to break part of the C=O bonds even at room temperature, and to release other nonisotopic CO_2_ molecules with ^12^C and/or ^16^O atoms. Only the Ru/Ce25PrO_*x*_ catalyst shows certain CO_2_ (^13^C^18^O^18^O; *m*/*z* 49) release at 30 °C, evidencing the poorest interaction with the gas-phase CO_2_ molecules.

The release of nonisotopic CO_2_ (^12^C^16^O^16^O; *m*/*z* 44) is relevant for all catalysts at 30 °C, and the percentage of ^12^C^16^O^16^O ranges between 22 and 34% of total carbon species, depending on the catalyst. These high percentages of evolved nonisotopic CO_2_ (*m*/*z* 44) indicate that the chemisorption of new carbon species upon the CO_2_ (^13^C^18^O^18^O; *m*/*z* 49) pulse leads to desorption of CO_2_ molecules previously chemisorbed on the catalysts. This process does not involve breaking C=O bonds necessarily.

The emission of ^12^C^16^O^18^O (*m*/*z* 46) is also relevant for all catalysts at 30 °C, and this species comes from the incorporation of one ^18^O atom of the CO_2_ pulsed (^13^C^18^O^18^O; *m*/*z* 49) to a previously chemisorbed ^12^C-containing surface species. On the contrary, the emission of ^12^C^18^O^18^O (*m*/*z* 48) is not relevant at 30 °C nor at higher temperatures for any catalyst, indicating that the two ^18^O atoms of a ^13^C^18^O^18^O molecule do not react with the same surface ^12^C-containing species.

The *m*/*z* 47 (^13^C^16^O^18^O) and *m*/*z* 45 (^13^C^16^O^16^O) signals indicate that the ^13^C atoms of the pulsed CO_2_ molecules are reoxidized with catalyst ^16^O once the ^13^C=^18^O bonds are broken, leading to the exchange of one or two oxygens, respectively. At room temperature, the double exchange prevails in the Ru/CeO_2_ catalyst (Figure 6a), and single exchange (Figure 6b and c) is more relevant in Pr-containing catalysts; the higher the Pr loading, the worse is the double exchange. This difference between Pr-free and Pr-containing catalysts suggests that the C=O bond-breaking rate is the slowest step of the process that involves: (i) CO_2_ adsorption, (ii) C=O bond breaking, (iii) reoxidation of surface carbon species with catalyst oxygen, and (iv) CO_2_ desorption. The slowest step (C=O bond breaking) is faster for Ru/CeO_2_ than for Ru/CeXPrO_*x*_ catalysts, and for this reason the Pr-free catalyst has more time to exchange both oxygen atoms of the CO_2_ molecule than the Pr-containing catalysts before the CO_2_ molecule is desorbed. It is known that the most active sites for CO_2_ dissociation are oxygen vacancies located at the Ru–CeO_2_ interface,^41,46^ and the negative effect of Pr in the exchange of oxygen atoms between catalyst and CO_2_ is in accordance with the negative effect of Pr in the Ru–CeO_2_ interaction.

Minor differences in the distribution of the carbon species are noticed between 30 and 100 °C, but significant changes are observed at 200, 250, and 300 °C. The main change occurring at 200 °C is that the percentages of evolved nonisotopic CO_2_ (*m*/*z* 44) drop while the percentages of double oxygen exchanged CO_2_ (*m*/*z* 45; ^13^C^16^O^16^O) increase, that is, the exchange of oxygen atoms of the chemisorbed CO_2_ molecules is enhanced. This means that the slow step of the oxygen exchange process (C=O bond breaking) at 30 and 100 °C is significantly enhanced at 200 °C. As mentioned, the most active sites for CO_2_ chemisorption and dissociation are vacant sites located at the Ru–CeO_2_ interface,^41^ and an increase in the reduction degree of the catalyst surface would promote a higher concentration of oxygen vacancies, which would explain the enhanced CO_2_ dissociation at 200 °C.

At this temperature, the presence of Pr favors the double exchange of oxygen, and the drop in *m*/*z* 44 signal (^12^C^16^O^16^O) and increase of the *m*/*z* 45 one (^13^C^16^O^16^O) are the highest for Ru/Ce25PrO_*x*_ and the lowest for Ru/CeO_2_. This could be related to the improved oxygen mobility into the ceria lattice upon Pr doping, as observed in H_2_-TPR. Once a ^13^C=^18^O bond of a ^13^C^18^O^18^O molecule is broken on a vacant site at the Ru–CeO_2_ interface, the created ^18^O species moves through the CeO_2_ support, leaving the vacant site available for further dissociative chemisorption of CO_2_. As such, the CeO_2_ support is a sink for ^18^O species, and the Pr doping improves the exchange of the oxygen atoms of the CO_2_ molecules by the oxygen of the catalyst due to the higher oxygen mobility.

For all catalysts, under the experimental conditions of the isotopic experiments, the release of CH_4_ species occurs in a significant percentage at 250 and 300 °C, and the formation of CH_4_ (*m*/*z* 15 for ^12^CH_4_ and *m*/*z* 16 for ^13^CH_4_) occurs together with the drop of the *m*/*z* 45 (^13^C^16^O^16^O) and *m*/*z* 46 (^12^C^16^O^18^O) signals. This means that, at 250 and 300 °C, the energy is high enough to hydrogenate the carbon intermediates once the ^13^C^18^O^18^O molecules are chemisorbed and the ^13^C=^18^O bonds are broken.

The percentages of CH_4_ species evolved (*m*/*z* 15 or *m*/*z* 16) and CO_2_ species depleted (*m*/*z* 45 and *m*/*z* 46) with regard to lower temperatures depend on the catalyst and on the temperature. As such, for Ru/CeO_2_ at both 250 and 300 °C, the main CO_2_ species consumed is ^12^C^16^O^18^O (*m*/*z* 46) and the main CH_4_ species evolved is ^12^CH_4_ (*m*/*z* 15). This is evidence that, for this catalyst, the species hydrogenated to CH_4_ are carbon intermediates present on the catalyst surface before the ^13^C^18^O^18^O pulse. The behavior of Ru/Ce3PrO_*x*_ is quite similar to that of Ru/CeO_2_, and only a few ^13^CH_4_ (*m*/*z* 16) species were detected at 300 °C. On the contrary, for Ru/Ce25PrO_*x*_ at 250 °C, the main CH_4_ species evolved is ^13^CH_4_ (*m*/*z* 16), and both the ^13^C^16^O^16^O (*m*/*z* 45) and ^12^C^16^O^18^O (*m*/*z* 46) signals drop. In this case, the hydrogenated carbon atoms come from the pulsed ^13^C^18^O^18^O molecules, which are hydrogenated once the ^13^C=^18^O bonds are broken. The distribution of carbon products changes at 300 °C, and the percentages of carbon species are the same for Ru/Ce3PrO_*x*_ and Ru/Ce25PrO_*x*_ at this temperature.

As a summary of the isotopic experiments, a dynamic equilibrium has been observed between gas-phase CO_2_ molecules and carbon species on the catalyst surface. At low temperature (30 and 100 °C), the adsorption–desorption of the CO_2_ molecules occurs both without breaking the C=O bonds and with breaking these bonds and exchanging oxygen atoms between the CO_2_ molecules and the catalysts. At 200 °C, the dissociation and exchange of oxygens is significantly favored with regard to the associative adsorption–desorption, and at 250 and 300 °C, surface intermediates created upon CO_2_ dissociation are hydrogenated to CH_4_.

Pr-doping on the CeO_2_ support has different effects in these processes. Pr hinders the Ru–CeO_2_ interaction and therefore hinders the dissociation of the CO_2_ molecules at low temperature, which takes place at the Ru–CeO_2_ interface. However, once the temperature is high enough (200 °C), Pr improves the oxygen mobility in CeO_2_, and this enhances the dissociation of the C=O bonds because the remaining oxygen atoms are delivered faster to the support sink, cleaning up the vacant sites.

### 3.5 In Situ Raman Spectroscopy Experiments

**Figure 7:**
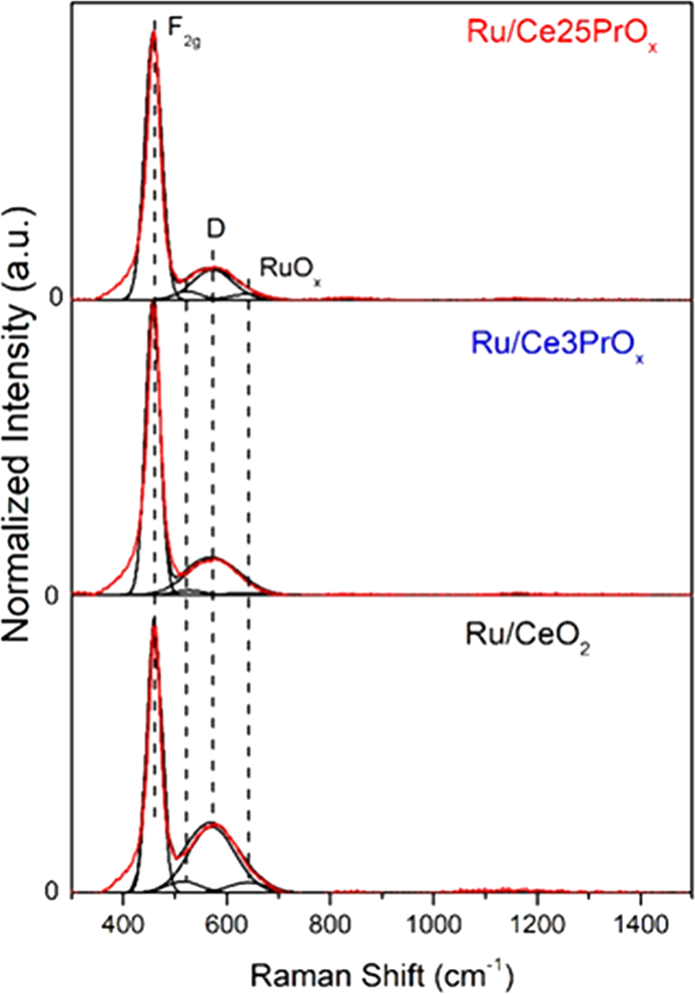
In situ Raman spectra recorded at 25 °C under methanation mixture (10% CO_2_, 40% H_2_, and N_2_ balance) after reduction at 450 °C with H_2_/N_2_ for 1 h.

The important role of catalyst vacant sites in the CO_2_ methanation reaction pathway has been inferred from isotopic experiments because these sites are related with CO_2_ dissociation and with oxygen mobility in the ceria support. Raman spectroscopy is a powerful tool to identify vacant sites on ceria, and in situ experiments have been performed to monitor the behavior of such vacant sites under reaction conditions. Figure 7 shows, as a representative example, the Raman spectra recorded for the three catalysts at 25 °C under the methanation gas mixture (10% CO_2_, 40% H_2_, N_2_ balance) in steady state after reduction at 450 °C in 50% H_2_/N_2_ for 1 h.

**Figure 8:**
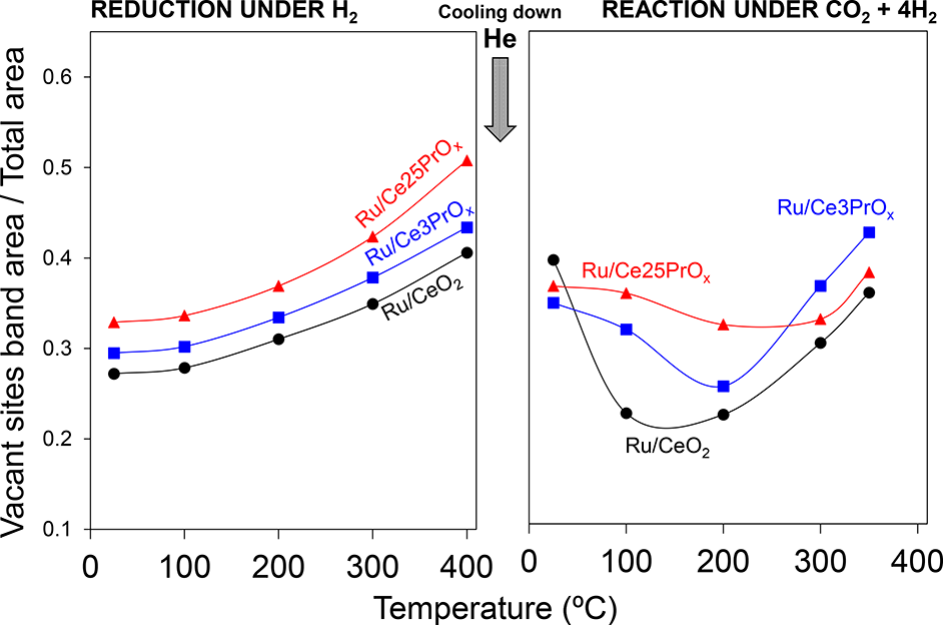
Evolution of the oxygen vacancy sites on CeO_2_ during the reduction pretreatent (50% H_2_/N_2_) and further heating under methanation mixture (10% CO_2_, 40% H_2_, N_2_ balance) monitored by Raman spectroscopy.

All of the spectra show the same characteristic bands, but the area and intensity of these bands differ in each catalyst and temperature. The main band around 465 cm^–1^ is assigned to the F_2g_ mode of the fluorite structure of ceria, and it is produced by oxygen anions breathing around the equilibrium position in the tetrahedral sites of the cubic unit cell. Another relevant band, denoted as D, appears around 565 cm^–1^, which is assigned to oxygen vacancies created upon removal of part of the ceria oxygens due to the partial reduction of Ce^4+^ and Pr^4+^ cations.^50−53^ The bands at 523 and 640 cm^–1^ are assigned to monocrystalline RuO_*x*_, and they strongly overlap with the isolate surface oxygen vacancy bands from the CeO_2_ lattice and the shoulder of 2TO overtone that results in a flattened band.^54−56^ To monitor changes in the oxygen vacancies population during the methanation reaction, all spectra have been deconvoluted, and the relative concentration of vacancies has been calculated for each spectrum as the ratio between the area of the vacant sites D band and the total area of the spectrum. The results of this quantification have been plotted in Figure 8 for the three catalysts during the reduction pretreatment and during further methanation experiments.

As expected, the number of vacant sites increases during the reduction pretreatment with H_2_, and Pr favors the creation of vacancies; the higher the Pr loading, the higher is the amount of oxygen vacancies. Partial reoxidation of these oxygen vacancies takes place by CO_2_ once the methanation mixture is fed to the reactor, and the reoxidation rate and degree are different for each catalyst. For Ru/CeO_2_, reoxidation occurs in a relevant extent at 100 °C, while reoxidation of oxygen vacancies increases progressively from room temperature until 200 °C for Ru/Ce3PrO_*x*_ and Ru/Ce25PrO_*x*_. This filling of oxygen vacancies observed by Raman spectroscopy between room temperature and 200 °C is consistent with the dynamic equilibrium between the gas phase and chemisorbed CO_2_ molecules deduced from isotopic experiments.

The amount of oxygen vacancies increases under methanation conditions above 200 °C (Figure 8), that is, the effect of H_2_ reduction is observed in the CO_2_ + H_2_ mixture. Figure 8 profiles evidence that, under methanation conditions (*T* ≥ 200 °C and CO_2_ + H_2_ gas mixture), each catalyst reaches a balance for each temperature between the H_2_ reduction and CO_2_ oxidation processes, and the amount of oxygen vacancies is the result of this balance. The highest concentration of oxygen vacancies is achieved by the Ru/Ce3PrO_*x*_ catalyst under CO_2_ methanation conditions, and this is also the most active catalyst (Figure 1) above 250 °C. This correlation suggests that Ru/Ce3PrO_*x*_ is able to keep the ceria support more reduced under methanation conditions than Ru/CeO_2_ and Ru/Ce25PrO_*x*_, and this has a positive effect on the catalytic activity because the chemisorption and dissociation of CO_2_ are very effective on reduced sites at the Ru–CeO_2_ interface.

These Raman spectroscopy experiments confirm the double role of Pr doping. On the one hand, Pr improves the reducibility of ceria, which is evidenced during the reduction pretreatment by creation of oxygen vacancies on the ceria support. On the other hand, Pr hinders the Ru–CeO_2_ interaction, which negatively affects the dissociation of CO_2_ and the reoxidation of oxygen vacancies, which is the most relevant event under the methanation gas mixture at temperatures below 200 °C. Once the temperature is high enough (*T* ≥ 200 °C), the two effects of Pr doping contribute in opposite ways to ceria reduction by H_2_ and reoxidation by CO_2_, reaching an optimum for the Ru/Ce3PrO_*x*_ catalyst.

### 3.6 In Situ DRIFTS Experiments

**Figure 9:**
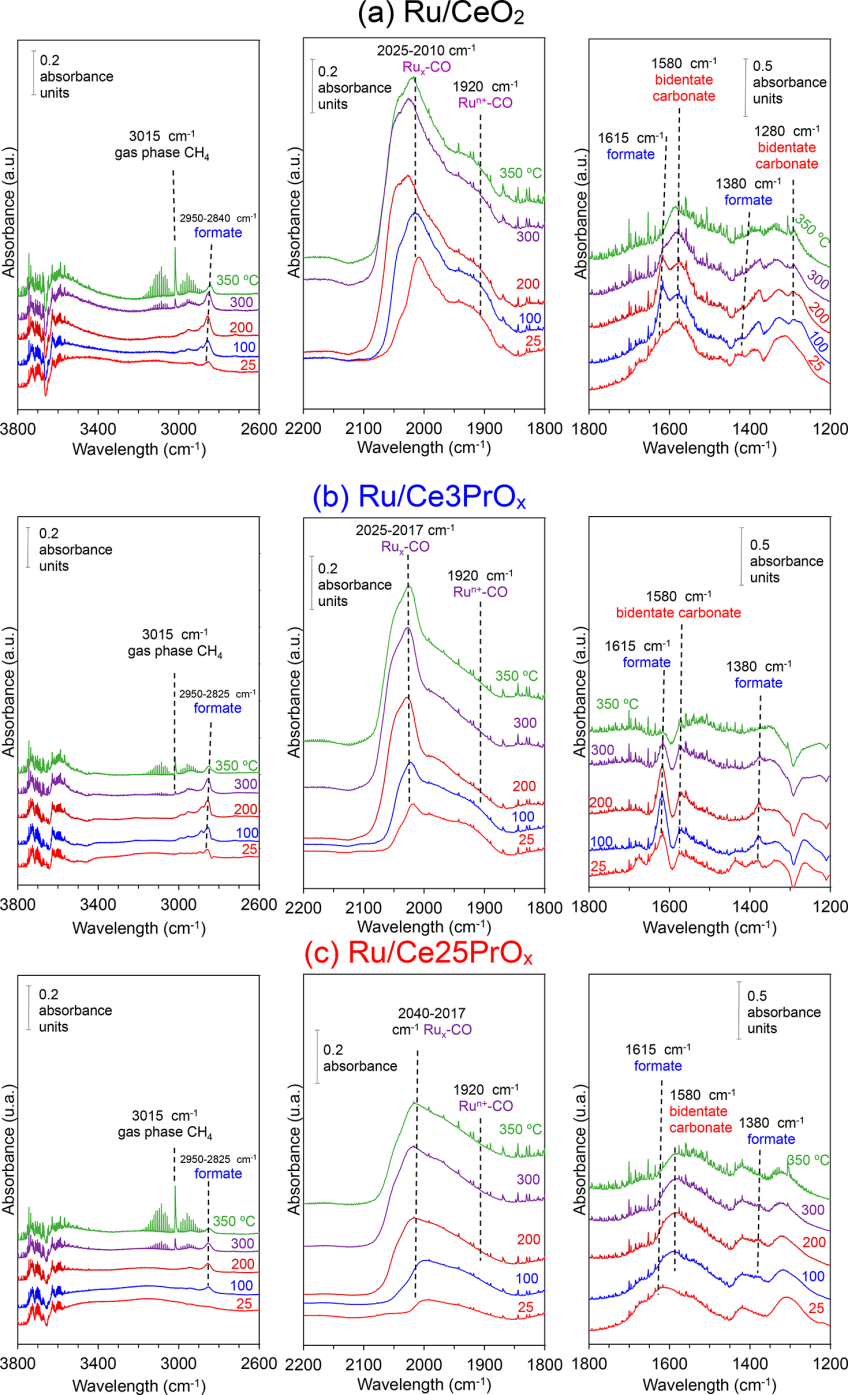
In situ DRIFTS spectra recorded in steady-state conditions under 10% CO_2_, 40% H_2_, and He balance for (a) Ru/CeO_2_, (b) Ru/Ce3PrO_*x*_, and (c) Ru/Ce25PrO_*x*_. Pretreatment at 450 °C under 50% H_2_/He.

Finally, the CO_2_ methanation reaction intermediates were studied by in situ DRIFTS measurements under the CO_2_ methanation reaction mixture at steady-state conditions, after reduction of the catalysts at 450 °C in H_2_/He for 1 h and cooling down to room temperature in He flow. A background spectrum was recorded at room temperature in He and subtracted from further spectra; therefore, bands shown in the spectra (Figure 9) only belong to surface species created (or depleted) under the CO_2_ methanation mixture. As shown in Figure 9, three relevant wavelength ranges were distinguished with characteristic bands assigned to C–O and C=O vibration modes (at 1800–1200 cm^–1^ range), ruthenium carbonyls (at 2200–1800 cm^–1^ range), and C–H vibration modes (at 2600–3200 cm^–1^ range), providing evidence of the formation of bidentate carbonates (at 1580 and 1280 cm^–1^), formates (2825–2950, 1615, and 1380 cm^–1^), and ruthenium carbonyls (1920 and 2017–2040 cm^–1^).

Pr affects the intensity of the signals of the different intermediates. The Ru/CeO_2_ catalyst shows bands compatible with the formation of bidentate carbonates, formates, and carbonyls, even at room temperature. These species are the same ones previously detected in similar conditions for Ru/CeO_2_ catalysts, and the changes introduced by praseodymium are related with the intensity of the signals, which are lower for the Ru/CeXPrO_*x*_ catalysts with regard to Ru/CeO_2_.

**Figure 10:**
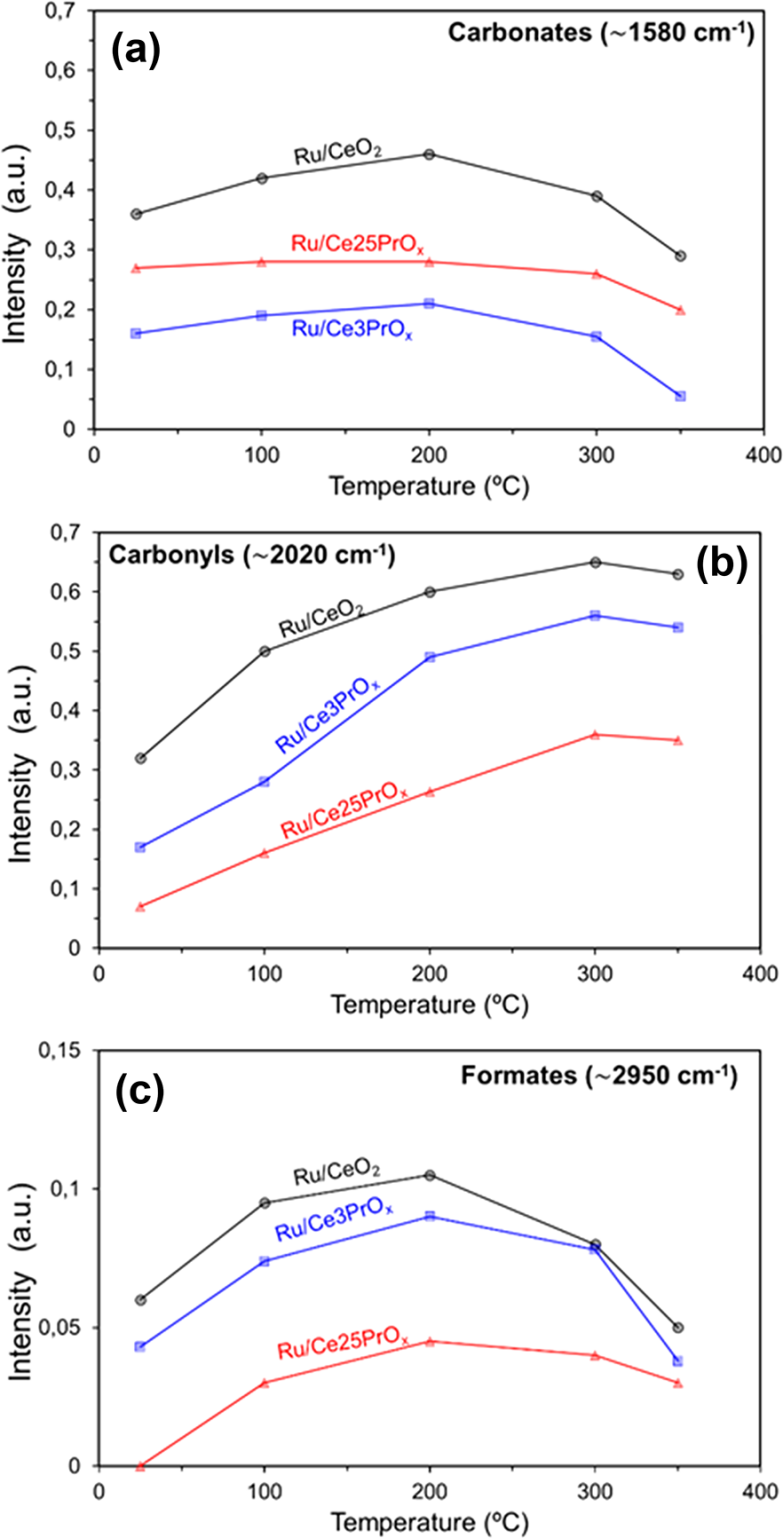
Evolution of (a) carbonate, (b) ruthenium carbonyl, and (c) formate signals with temperature during in situ DRIFTS experiments under 10% CO_2_, 40% H_2_, and He balance (pretreatment in H_2_/He at 450 °C).

The presence of carbonates, formates, and carbonyls on the catalysts surface at room temperature is consistent with the conclusions of the isotopic exchange experiments, where evidence about the dynamic equilibrium between gas-phase CO_2_ and surface carbon species was found, and is also in agreement with the reoxidation of the catalysts by CO_2_ deduced from Raman spectroscopy experiments. As a general trend, the intensity of the signals increases with temperature until a maximum value, and above this maximum the signals remain stable or decrease. For easier analysis of changes with temperature, Figure 10 shows the intensity of selected bands for each species identified in the spectra after baseline subtraction.

It is reasonable to assume, according to Figure 10, that carbonates are hardly hydrogenated to methane while formates and carbonyls are the most efficient reaction intermediates toward total hydrogenation. Ru/CeO_2_ support doping with Pr diminishes the formation of carbonates, as observed in Figure 10a, therefore keeping the catalyst surface with a lower coverage of carbon species with poor relevance for CH_4_ production. This effect is expected to have a positive contribution to the catalytic behavior and could be related with the improved ceria reduction by Pr doping, as deduced in H_2_-TPR characterization. It is known that carbonates are created on ceria after chemisorption of CO_2_ on surface oxygens, and the improved reducibility of the support will decrease the amount of these types of chemisorption sites under the methanation reaction conditions.

The surface concentrations of carbonyls (Figure 10b) and formates (Figure 10c) also decrease for Pr-containing catalysts with regard to Ru/CeO_2_; that is, Pr not only hinders the formation of unproductive carbonates but also hinders the formation of the productive reaction intermediates, and this is expected to have a negative effect on the catalytic activity. Once again, DRIFTS experiments show a double role of Pr affecting the formation of carbon surface species that are both productive and unproductive for methane formation.

It is important to compare the trend of the carbonyl (Figure 10b) and formate (Figure 10c) signals with temperature. The surface coverage of formates increases from room temperature until 200 °C for all catalysts and decreases above this temperature once the production of methane starts to be relevant. This is consistent with the participation of formates as reaction intermediates. On the contrary, carbonyl concentrations increase with temperature until reaching a quite stable value, and this could indicate that the slowest reaction step is the hydrogenation of carbonyls to methane.

In conclusion, the DRIFTS experiments show that Pr doping decreases the population of surface carbon species created on the catalysts upon CO_2_ chemisorption under methanation reaction conditions, affecting both productive reaction intermediates (formates and carbonyls) and unproductive carbonates.

## 4 Conclusions

The effect of Pr in Ru/CeO_2_ catalyst for CO_2_ methanation has been analyzed in this study, and the main conclusions can be summarized as follows:

Pr doping in low concentration is beneficial for CO_2_ methanation, and it has been observed that a 3%-doped Ru/CeO_2_ catalyst is moderately more active than bare Ru/CeO_2_ from 250 °C onward. On the contrary, high Pr concentration has a negative effect on the catalytic activity.

H_2_-TPR experiments showed that Pr doping has a double role in the reducibility of Ru/CeO_2_. Pr hinders the reduction of ruthenium species because it partially impedes the Ru-CeO_2_ interaction, but it improves the reducibility and oxygen mobility of the ceria support.

Pulse experiments with isotopic CO_2_ evidenced a dynamic equilibrium between gas-phase CO_2_ molecules and carbon species on the catalyst surface. At low temperature (30 and 100 °C), the adsorption–desorption of the CO_2_ molecules occurs both without breaking the C=O bonds and with breaking these bonds and exchanging oxygen atoms between the CO_2_ molecules and the catalysts. At 200 °C, the dissociation and exchange of oxygens is significantly favored with regard to the associative adsorption–desorption, and at 250 and 300 °C the surface intermediates created upon CO_2_ dissociation are hydrogenated to CH_4_. Pr doping on the CeO_2_ support has different effects in these processes. Pr hinders the Ru–CeO_2_ interaction and therefore hinders the dissociation of the CO_2_ molecules at low temperature, which takes place at the Ru–CeO_2_ interface. However, once the temperature is high enough (200 °C), Pr improves the oxygen mobility in CeO_2_, and this enhances the dissociation of the CO_2_ molecules because the remaining oxygen atoms are delivered faster to the support sink and the dissociation sites are cleaned up faster. In addition, once the methanation temperatures are achieved (*T* > 200 °C), hydrogenation of the previously chemisorbed surface carbon species is more favorable for Ru/CeO_2_, while Pr doping favors the hydrogenation of just chemisorbed CO_2_ molecules.

In situ Raman spectroscopy experiments confirmed the double role of Pr doping and showed that Pr improves the reduction of ceria by H_2_, with creation of more oxygen vacancies on the Pr-doped ceria supports than in bare ceria. Nevertheless, Pr hinders the Ru–CeO_2_ interaction, and this negatively affects the dissociation of CO_2_ and the reoxidation of oxygen vacancies, which is the most relevant event under the methanation gas mixture at temperatures below 200 °C. Once the temperature is high enough (*T* ≥ 200 °C), the two effects of Pr doping contribute in opposite ways to ceria reduction by H_2_ and reoxidation by CO_2_, reaching an optimum for 3% Pr doping.

Finally, in situ DRIFTS experiments evidenced that Pr doping decreases the population of surface carbon species created on the Ru/CeO_2_ catalysts surface upon CO_2_ chemisorption under methanation reaction conditions, affecting both productive reaction intermediates (formates and carbonyls) and unproductive carbonates.
